# Development and Evaluation of e-CA, an Electronic Mobile-Based Food Record

**DOI:** 10.3390/nu9010076

**Published:** 2017-01-18

**Authors:** Sophie Bucher Della Torre, Isabelle Carrard, Eddy Farina, Brigitta Danuser, Maaike Kruseman

**Affiliations:** 1 Department of Nutrition and Dietetics, School of Health Sciences-Geneva (HEdS-GE), University of Applied Sciences and Arts Western Switzerland (HES-SO), Rue des Caroubiers 25, 1227 Carouge, Switzerland; isabelle.carrard@hesge.ch (I.C.); eddy.farina@hesge.ch (E.F.); maaike.kruseman@hesge.ch (M.K.); 2 Institute for Work and Health, University of Lausanne, Route de la Corniche 2, 1066 Epalinges-Lausanne, Switzerland; brigitta.danuser@hospvd.ch

**Keywords:** mobile food record, technology-based assessment of dietary intake, nutritional epidemiology, development and evaluation

## Abstract

Measures that capture diet as validly and reliably as possible are cornerstones of nutritional research, and mobile-based devices offer new opportunities to improve and simplify data collection. The balance between precision and acceptability of these data collection tools remains debated, and rigorous validations are warranted. Our objective was to develop and evaluate an electronic mobile-based food record for a research setting. We developed e-CA, which includes almost 900 foods and beverages classified in 14 categories and 60 subcategories. e-CA was evaluated using three different methods: (1) usability and acceptability through a logbook and qualitative interviews; (2) dietary intake accuracy through comparison with 2 unannounced 24-h phone recalls on overlapping days; and (3) reliability and process comparison with a paper-based food record in a laboratory setting with a randomized design. e-CA proved to be intuitive and practical and was perceived as modern, trendy, and fun. Comparisons of e-CA with 24-h telephone recalls or paper-based food records in a laboratory setting with two small convenient samples showed good agreement but highlighted the well-known difficulty of estimating portion sizes and a necessary learning time to use the app. e-CA is a functional tool that has the potential to facilitate food intake measurement for research by increasing the pleasure of using the food record tool and reducing the perceived burden for the participants. It also decreases the workload, costs and the risk of transcription errors for researchers.

## 1. Introduction

Measures that capture diet as validly and reliably as possible are the basis of nutritional research, and mobile devices offer the opportunity to improve and simplify data collection [1]. Food frequency questionnaires (FFQs), 24-h recalls (R24s), food records, and interviews are the tools most commonly used for dietary intake assessment in nutritional research or epidemiology [2]. Each method has its own limitations, affecting data collection and weakening the conclusions of research in the field of nutrition, especially if errors in reporting are not addressed to the extent possible using appropriate tool selection and statistical techniques [1]. One major limitation of the FFQ and the R24 is their reliance on memory. Investigator-led interviews can induce bias in participants’ answers. Food records do not rely on memory but induce a high burden on participants, affecting their motivation and the quality of the data. Moreover, this method requires a substantial amount of work for the investigator to decipher and transcribe subjects’ records, with a high risk of errors and time-related costs [3].

New technologies may provide interesting improvement opportunities for traditional food intake measures. Ilner et al. reviewed 74 studies using innovative technologies aimed at improving the food intake measures in nutritional epidemiology. They concluded that, even if methodological features of new technologies and conventional methods often overlap, new technologies might enhance dietary assessment through more cost- and time-effective data collection and higher subject acceptance [4]. The need for efficient tools for large epidemiological studies has encouraged the development of software for self-reported online R24s or checklists, such as ASA24 [5], myfood24 [6], Nutrinet [7], and others [8,9,10,11]. These tools have shown good agreement with an interviewer-administered R24 [5,7] and with true intake [5]. For studies necessitating food records, personal digital assistants (PDAs) and smartphones can be used as electronic food diaries or as photography-based records [12]. Electronic food records allow real-time recording, facilitate recording when eating out of home, and, therefore, could reduce memory bias. They may also reduce burden on participants and thus increase adherence and satisfaction. These devices may also decrease the investigators’ workload, risks of errors, and costs by directly recording the raw consumption data. Numerous commercial smartphone applications are available for tracking food intake, but they are mostly designed to support behavioral changes. Therefore, they provide feedback to the users, who may modify their behavior, rendering these applications incompatible with research objectives. Photography-based food records are promising but still require significant involvement of a trained investigator for data analysis, at least until sufficient technological advances allow full automation of this process [13]. For now, a simple-to-use electronic food record is still warranted. Therefore, our objective was to develop and evaluate a mobile application, called e-CA (electronic *carnet alimentaire*, “food record” in French), that would facilitate food and beverage tracking for research participants and for researchers through ease and comfort of use while maintaining a comparable performance with traditional tools.

## 2. Materials and Methods

**Figure 1:**
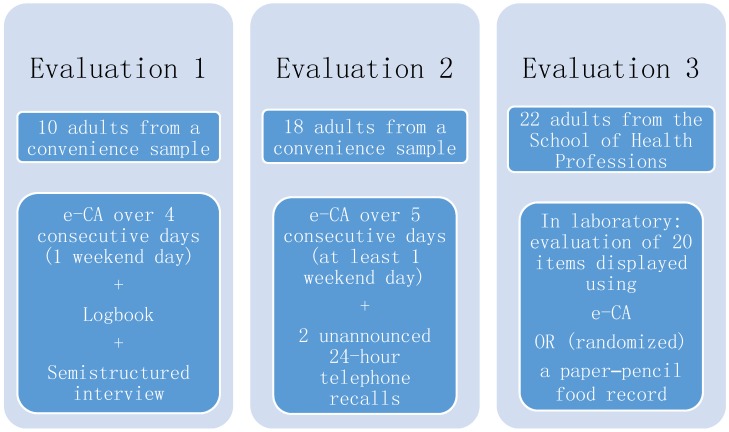
Illustration of the three steps for evaluating the electronic food record e-CA.

After developing e-CA in a multistage process, the tool was evaluated using three different methods (Figure 1): (1) usability and acceptability through a logbook and qualitative interviews; (2) dietary intake accuracy through comparison with two unannounced R24s on overlapping days; and (3) reliability and process comparison with a paper-based food record in a larger sample with a randomized design. After each step, the application was adapted accordingly.

The Geneva Cantonal Ethics Committee on Research Involving Humans reviewed and approved this study (project 14-261). All participants signed informed consent forms.

### 2.1. Development and Description of e-CA

e-CA is a Web-based electronic food record that was designed to be used on a smartphone but can also be used with any Internet connected device, such as a tablet or computer. The tool includes an online researcher platform for creating and managing participants’ accounts and uploading participants’ data. Specifications regarding technical issues, structure of the app, and interface between the app and website were chosen on the basis of a literature review on existing tools [9,14,15,16] and created in collaboration with two software developers. Special attention was paid to limit the number of screens when entering a food, to avoid long lists for scrolling through, and to provide a generic intuitively categorized food list.

**Figure 2:**
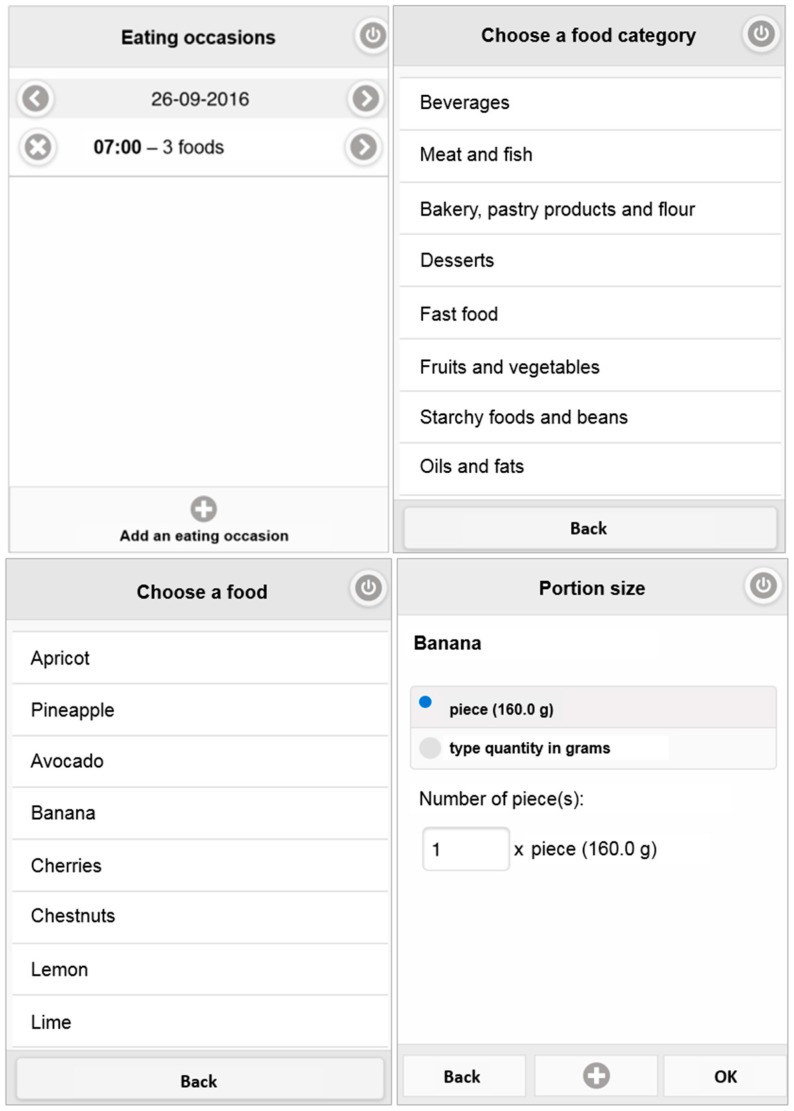
Screenshot illustrating the 3 steps for using e-CA: (1) creating an “eating occasion” (first picture); (2) selecting a food or drink from a category and subcategory (second and third pictures); and (3) defining the size of the portion consumed (fourth picture).

When using e-CA, users follow a three-step process (Figure 2): (1) creating an “eating occasion” each time they eat or drink something, specifying the date and time of day; (2) selecting the foods and/or drinks consumed from the list of categories and subcategories; and (3) defining the size of the portion consumed by either selecting from the proposed portion sizes and indicating the number of portions consumed or by entering the quantity (in g or mL). e-CA allows the registration or modification of any eating occasion earlier in the day or on a previous day. e-CA also offers the opportunity for users to write a comment for each eating occasion (e.g., list a food item lacking in the database, clarify a recipe, or indicate a special event). This feature was added after the first step of the evaluation.

The database includes almost 900 food and beverage items hierarchically organized into 14 categories and 60 subcategories. Based on a review of the literature, consultations with dietitians experienced in nutritional epidemiology, and several visits to traditional and virtual supermarkets, items were classified in a consumer-oriented way, as opposed to classic nutritional classification. For example, the “breakfast” category includes items usually consumed for breakfast in Switzerland. Some foods can be found in several categories, making them easier to locate. For example, “butter” can be found in three categories: “fats”, “dairy products”, and “breakfast”. To limit the number of options and facilitate the choice process, most of the foods and beverages are generic ones. Similarly, we chose an intermediate level of detail; for example, yogurts have two levels of descriptors, proportion of fat and presence or not of added sugar, but there is no information about the flavor. For the portion sizes, one to three common options were included for each item. For example, in Switzerland, yogurts usually come in 125 g, 150 g, or 180 g containers. For fruits, a medium and a large portion were proposed. For vegetables or starches, the options correspond to the amount on the plate (e.g., 1/3 of the plate of carrots =120 g).

The administrative platform on the website permits the creation of user accounts by defining a login and password. The researchers have to send this access information as well as an Internet link to participants in order for them to log into e-CA. The output of the app is a spreadsheet including the structured list of consumed foods and beverages, together with their weight and the time of consumption. At present, there is not yet a link with a nutritional database to calculate energy and nutrient intakes.

### 2.2. Evaluation 1: Usability and Acceptability

The first step of the evaluation aimed to assess the usability and acceptability of the tool in a heterogeneous convenience sample of 10 adults (3 men, 7 women; 1 was 20–29 years old, 4 were 30–39 years old, and 5 were 40–60 years old). Among them, two were dietitians and at least 5 had no experience with food records. Participants received the instruction to enter all foods and beverages consumed over 4 consecutive days, including one weekend day. They received a brief oral training and a paper document on features and functionalities of the app. During that time, participants also filled in a paper-based logbook, stating any problem or bug they encountered. At the end of the period, semi-structured interviews were conducted to investigate the ease of or barriers to the use of e-CA, its strengths and weakness, the perceived workload, the errors related to e-CA’s system, the circumstances of use, and any suggestions for improvement. The interviews were recorded and transcribed. We conducted a descriptive content analysis of the qualitative data [17] to identify salient themes.

### 2.3. Evaluation 2: Comparison with R24s

In order to evaluate the accuracy in terms of energy, macronutrient, and food group intake, the second evaluation included a comparison of the application with the method of multiple pass telephone R24s. A convenience sample of 21 participants (8 were 20–29 years old, 8 were 30–39 years old, and 5 were 40–60 years old) was recruited, from which 18 (12 men, 9 women) used e-CA for 5 consecutive days, including at least one weekend day. All received brief oral training and a paper document on the features and functionalities of e-CA. During that time, trained dietitians performed 2 unannounced R24s. Each participant had received a booklet with illustrations of the portion sizes of several foods [18] to be used during the R24s. Mean energy and macronutrient intakes were calculated for each day, separately for each method, e-CA and R24, using the software Prodi 6.3 (Nutri-Science GmbH). The total consumption of two food groups (dairy, fruit and vegetables) was calculated in servings per day, according to the Swiss food guide pyramid [19]. The results obtained with e-CA were compared with the results obtained on the corresponding day through R24 using the Wilcoxon signed-rank test. Since the distribution of the variables was not normal, nonparametric tests were used. A Bland-Altman analysis was used to assess the agreement between the two methods by studying the difference between e-CA and R24 compared to the average intake of these measurements on overlapping days [20]. As described by Bland and Altman, the limits of agreement were set as two standard deviations (SDs) of the difference above and below the mean difference [21].

### 2.4. Evaluation 3: Comparison with Paper-Based Food Records

The third step of the evaluation consisted of a comparison between e-CA and a paper-based food record in a laboratory setting. For this, 20 preweighed food items representing 3 meals and 2 snacks were displayed: breakfast (chocolate cereals, bread with butter and jelly), snack 1 (coffee, cereal bar), lunch (sliced chicken in creamy sauce, rice, carrots, apple, chocolate), snack 2 (tea, cookies), and dinner (vegetable soup, cheese quiche, green salad, grapes). A convenience sample of 22 participants (7 men, 15 women; 12 were 18–24 years old, 5 were 25–39 years old, and 5 were 40–60 years old) was randomly assigned to individually evaluate the items on display using either e-CA or an open-text-field paper-based food record after a brief training. Participants were students or collaborators of the School of Health Sciences Geneva but were external to the Nutrition and Dietetics Department.

Outcomes of interest were (1) naming of foods and beverages; (2) portion size estimation; and (3) workload for the investigators to analyze the records. We used the method developed by S.I. Kirkpatrick et al. [5] to compare the terms used by participants in both groups to the reference described *a priori*. For each of the 20 items, an experienced dietitian compared the terms used by participants and classified them as an exact or best match (e.g., “pumpkin soup” vs. “vegetable soup”), a close match (e.g., “brown bread” vs. “bread”), or a far match (e.g., “chocolate and cereal bar” vs. “crackers”). Items not on display that were recorded received the label *intrusion* and items on display but not recorded received the label *exclusion*.

Since portion sizes were established in e-CA but open to interpretation in the paper-based food record, we compared the exactitude of portion size evaluation by the participants for each instrument. Two dietitians who were not part of the project independently transformed all the portions from the paper-based food records into weights and entered the quantities of consumed food items recorded with both instruments into a food composition database. They recorded the time spent for entry and calculation of each record and documented each personal interpretation. We compared (1) the mean time to enter the record in the nutritional software depending on the type of instrument (average time of the two dietitians); (2) the percentage of error in portion size estimation depending on the type of instrument; and (3) the energy intake estimation depending on the type of instrument.

## 3. Results

### 3.1. Evaluation 1

The ten participants declared that e-CA was easy to use, intuitive, and practical. However, some training time was at first necessary for users to feel comfortable using the application and to find a food in the right category. Several participants highlighted that they appreciated not having to handwrite, which was considered a gain in terms of time. Interviews revealed that the food and beverages were logically classified but that some food items were missing. In addition, participants found it difficult or burdensome to enter composed or mixed foods (e.g., lasagna or pasta carbonara). This could have led to omissions. The evaluation of portion sizes was described as the most difficult part. Participants found the proposed standard portion sizes useful and helpful, but two of them would have liked pictures to help evaluate portion sizes. Two participants used their personal kitchen scale to weigh foods.

Four participants found that it took too long to enter each food and beverage. Some proposed adding a research tool to find foods more easily. Most participants estimated that daily input necessitated a total of 10 to 15 min; however, three participants spent a mean of 30 min per day. The participants entered foods and drinks in e-CA either as they went along, for each eating occasion (preferably when they were at home or when alone), or in a postponed manner (such as entering the data for the whole day in the evening). In summary, the main difficulties were portion size estimations, composed or mixed dishes, time to enter a food item, and some missing foods. Recommendations from the participants included adding a search tool to find foods and drinks more easily, asking for the place where food is consumed, adding the possibility of writing a comment, adding some missing foods, including pictures to help with portion size estimation, and improving the graphic design.

When asked if they would prefer a paper-based food record, the participants answered that they thought e-CA would be easier, more practical (always carried with them), more modern and trendy, and, mainly, more fun. Participants perceived this last aspect as the main added value of the app. After this first evaluation, missing foods were added in the food database, some foods were duplicated to facilitate navigation through categories, and a feature allowing users to write a comment for each eating occasion was added.

### 3.2. Evaluation 2

The second step of the evaluation showed that intakes measured by the application had good correlation with those performed by the two unannounced R24s conducted via phone.

Table 1 compares nutrient and food group intakes between the two methods. The comparison of the nutrient intakes showed that protein and carbohydrate intakes were similar for both methods and that there was a statistically significant difference of 10 g in lipid intake, with e-CA yielding the lower result. The related 100 kcal difference (−4.2%) between the two methods was not statistically significant. Regarding consumption of dairy and fruits and vegetables, the R24 method found a higher intake of, respectively, 0.2 and 0.3 servings per day. The large standard deviations reflect the variability of intakes between individuals.

**Figure 3:**
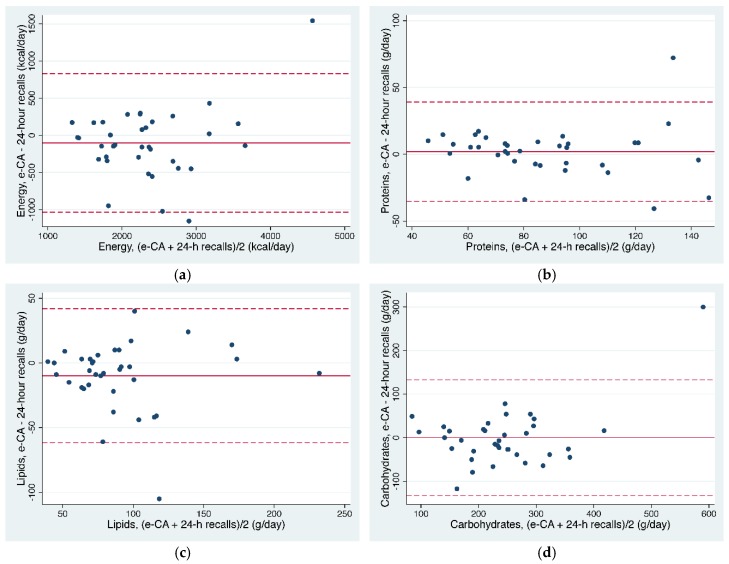
Bland-Altman plots of the mean difference (plain line) between e-CA and 24-h recall versus the mean intake of the two methods for energy (**a**), protein (**b**), lipid (**c**), and carbohydrate (**d**) intakes. The limits of agreement (dashed lines) equal 2 standard deviations above and below the mean difference

Figure 3 shows the Bland-Altman plot for the agreement between e-CA and the R24s for energy and macronutrient intakes by plotting the difference between the 2 methods (*y* axis) compared with the average results of both methods (*x* axis). Limits of agreement, defined as the mean difference ±2 standard deviations, ranged from −1036 to 832 kcal for energy, −35.3 to 39.2 g for proteins, −61.7 to 41.9 g for lipids, and −132.9 to 132.7 g for carbohydrates. One (for lipids and carbohydrates) or two (for energy and proteins) subjects over or underestimated their intake by greater than 2 SDs above the mean difference. In the food group analysis, two subjects were out of the limits of agreement for dairy and one subject for fruits and vegetables.

### 3.3. Evaluation 3

In a laboratory setting, the average time necessary to finish the task was 19 min with e-CA and 9 min with the paper-based food record. Participants using e-CA identified the items with more precision than those using the paper-based food record (77.3% vs. 60.0% of exact matches). However, the risk of exclusion (overlooking entering an item) was slightly higher with e-CA (6.8% vs. 5.5%) (Table 2). No intrusions were recorded.

Two dietitians unaware of the items displayed independently entered the outputs of both methods in a food composition database. Entering the e-CA output took significantly less time than completing the paper-based food records (mean time 8.2 min ± 3.1 vs. 11.8 min ± 2.4, *p* < 0.05).

Table 3 compares, for each food or drink item, the mean portion size estimated by the participants, using either e-CA or the paper food record, and analyzed by the two independent dietitians. Comparison with the real weight of each item shows a 3% overestimation for e-CA and a 3.9% and a 4.7% overestimation for each dietitian. The mean estimations fall within ±10% of the real weight for 7/20 items with e-CA and for 8/20 or 5/20 items with paper-based food records, depending on the investigator.

The foods and drinks displayed were equivalent to 2372 kcal. The mean estimations of the two dietitians were, respectively, 2150 kcal and 2090 kcal using e-CA, and 2300 kcal and 2043 kcal using paper-based food records. The estimations ranged between 1459 and 2932 kcal.

## 4. Discussion

e-CA is an electronic food record developed in order to facilitate the tracking of food and beverage consumption for research participants as well as for researchers. Ease, comfort of use, and comparable performance to traditional tools were the evaluation criteria. In two small convenience samples, this dietary assessment tool showed good agreement with R24s or paper-based food records and was considered by the users to be intuitive, practical, modern, and fun. Still, the evaluation highlighted the well-known difficulty of estimating portion sizes.

As smartphones become more and more popular and people become increasingly connected, technology-based dietary intake assessment is becoming inescapable. In this study the participants’ opinions showed that the pleasantness of e-CA counterbalanced the potential difficulties of its use. Hutchesson et al. evaluated the acceptability and accuracy of three different food record methods; online accessed via computer, online accessed via smartphone, and paper based [22]. Eighteen young women completed three 7-day food records in a random order. Most participants (89%) least preferred the paper-based records. Nine participants preferred computer, eight preferred smartphone, and only one preferred paper-based records. Similarly, for R24s, in a large sample (1081 adults), 70% of the respondents preferred a Web-based, automated, self-administered R24 (ASA24) to a standard interviewer-administered R24 following the automated multiple-pass method [23].

Recently, several online automated R24 systems have been developed, especially as part of large epidemiological studies [5,6,7,8,24]. Some of these tools may also be used as food records [6,7]. Electronic food records have also been developed, though more often as part of weight loss interventions, with the primary aim of self-monitoring [14,25]. However, food records remain an essential tool for research in nutrition, especially for investigating the structure of eating habits over several days.

Several existing tools include very large databases of food items (3000 to over 40,000), trying to be as exhaustive as possible [5,6]. Developers of My Meal Mate, a smartphone application supporting self-monitoring and weight loss, based its choices on focus groups and a pilot trial, which showed that people wanted to select the exact food they had eaten and that the food composition database was a limiting factor in engaging with the app [6,25]. On the other hand, we made the decision to reduce the database and to integrate more generic foods. The latest option has the advantages of simplifying data entry for participants by offering fewer options, contributing to a reduction in participant fatigue and requiring less maintenance to keep it up-to-date. Despite the limited list of foods, our results showed a comparable accuracy with tools including similar or larger databases. DietMatePro, a food record on PDAs, found no statistically significant difference between energy and macronutrient intakes when compared to an R24. In that study, energy intake was underestimated by 3.1% compared with an R24, whereas e-CA resulted in a 4.2% underestimation [14]. INTAKE24, an online R24 tool, was found to provide estimates of energy intake that were 1% lower on average than interviewer-led recall, with the limits of agreement ranging from −49% to +93%. For all macronutrients and micronutrients, mean intakes were within 10% of the interviewer-led recall [8]. In an adolescent population, Myfood24 underestimated energy intakes by 3% when compared with interviewer-led recalls, with the limits of agreement ranging from −39% to +34% [6]. The Bland-Altman test is increasingly used to compare agreement between two methods. In our study, the comparison of energy, macronutrient, and food group intake assessed by the application versus the intakes recorded by the R24 revealed good agreement between the two methods. Bland-Altman plots showed that the bias between the methods appeared consistent over the range of intakes. Other studies comparing a technology-based dietary assessment to an interview-led R24 using Bland-Altman plots found, as we did, rather large agreement limits [6,8,25].

The estimation of dietary intake remains challenging, and a comparison of paper-based food records with objective measures of energy expenditure or protein intake has indicated an underreporting of 4% to 37% [26]. In the third assessment of e-CA, we found a very large range of energy estimation for the same foods displayed. This can be explained by several factors: (1) forgetting to report some food items in e-CA or on the paper-based food record; (2) choosing a wrong description for the food; (3) under or overestimating the portion size of the food; and (4) variability between dietitians in interpreting the food records and entering the food items in the food composition database.

Portion size estimation represents a large source of error in food intake measurement [27], and self-reporting is especially problematic [4]. In e-CA, participants appreciated that we offered proposals for standard portions. This may help users to simplify data collection for usual preportioned food and accurately convert an objective portion into weight (for example, 1/3 of the plate of carrots). However, users may be tempted to choose one of the proposed portions even if their portion is very different. In our study we observed larger differences in the estimation of some portions between e-CA and paper-based food records, especially with drinks. For example, the 200 mL bowl of soup was recorded as 300 mL, which is the e-CA proposition. More generally, this system passes the responsibility of the interpretation of a descriptive portion from the researcher on to the participants and may create an illusion of precision. Food photographs improve portion size estimation [28], and several online R24 systems have integrated such features [6,7,8,24]. Also, some tools use participants’ pictures of their plate before and after meals [29,30]. For now, the quantification of portions requires a trained expert, but automated systems are rapidly emerging and may simplify this step in the future [31].

Electronic tools lead to substantial cost savings by asking users to collect dietary data. However, the lack of input from a professional dietitian who would usually point out missing information, forgotten items, or possible errors may decrease the accuracy of data. In our study, intakes of lipids differed between both methods (10 g per day). This error is likely attributable to the fact that the participants tended to forget to enter added fats into e-CA, while the dietitian systematically asked about this topic during the R24. When analyzing e-CA records, we noticed that salad dressing or oil used to grill was often missing. Condiments were also often forgotten. A way to avoid this would be to add prompts in the system, reminding users about condiments, dressings, or added fats [5]. Whereas prompts or pop-ups may improve the quality of the data, it is important to balance the researcher’s need to collect details with the risk of lengthening the assessment and annoying participants. Indeed, in a focus group study, Carter et al. found that participants declared they preferred a clean design with no pop-ups [6].

Compared to a paper-based food record, using an electronic tool requires additional time for learning and adaptation. As opposed to spontaneously describing the foods eaten, electronic food records require familiarization with the way foods are organized in the database. In the first evaluation step, seven participants reported needing 10 to 15 min per day to report for the whole day, whereas three participants spent an average of 30 min per day. This is comparable or even faster than other tools, such as DietMatePro [14], an electronic food record on PDAs, with which users reported taking an average of 8.5 min to record a typical meal, or My Meal Mate, a smartphone application designed to support weight loss, with which users reported taking 7 min to enter a meal or 22 min for the whole day [25]. Adherence is a recognized challenge, and the completion of a food record usually declines over time. The time participants agree to spend using a dietary assessment tool is limited. Carter et al. found, using focus groups, that adults were willing to spend 10 to 20 min to complete a dietary assessment, depending on the required frequency of use, but this time fell to 10 to a maximum of 15 min for adolescents [6].

In the present study, R24s were realized after participants had entered all food in e-CA. An overestimation of agreement between the two methods is therefore possible, as completing e-CA records may have impacted the accuracy of the following R24 by reducing memory bias. Also, the true intake remained unknown, and the portion proposed in e-CA may have influenced participants when they were interviewed for the R24. During the five days of electronic food recording, the two R24s were unannounced to get closer to real research conditions.

e-CA helps to avoid some pitfalls of paper-based food records, such as difficulties reading what participants have written, errors in transcription, or the fact that some participants, despite detailed instructions, lack precision in the description of the food they have eaten. Moreover, mobile-based electronic food records have the main advantage of facilitating data collection and management, reducing the burden on participants and researchers. However, food records, whether electronic or paper based, still have limits such as influencing behavior through real-time recording.

### Improvements, Perspectives

The next step will be to link e-CA to a national food composition database. This step should be facilitated by the fact that most food items are generic and few brand foods have been included in the app. This last point should also simplify the use of e-CA for contexts other than Switzerland. More generally, the simplicity of the technical structure of e-CA also enables future adaptations for specific settings; nevertheless, e-CA should be tested with diverse populations, such as adolescents or older adults. Some future developments should be evaluated and tested such as a search tool, pictures of standard portion sizes to help with estimation, alarms to help participants remember to fill in the electronic food record, or carefully selected prompts to limit the number of forgotten foods.

## 5. Conclusions

e-CA is a functional tool that has the potential to facilitate food intake measurement for research by increasing the pleasure of using the food record tool and reducing the perceived burden for the participants. Although it does not overcome intrinsic limitations of dietary assessment, it decreases the risk of transcription errors, workload, and costs for the researchers.

## Figures and Tables

**Table 1:** Paired comparisons (Wilcoxon signed-rank tests) of mean intake of nutrients, energy, and food groups measured with the e-CA app and two 24 h recalls.

Intakes	e-CA Mean (±SD)	24 h Recall Mean (±SD)	*p*-Value
Protein (g/day)	88 (±28)	86 (±29)	NS
Lipids (g/day)	86 (±41)	95 (±41)	*p* < 0.05
Carbohydrate (g/day)	244 (±113)	244 (±84)	NS
Energy (kcal/day)	2287 (±792)	2388 (±664)	NS
Fruit & vegetables (serv/day)	3.5 (±2.5)	3.9 (±2.3)	NS
Dairy (serv/day)	1.8 (±1.8)	2.1 (±1.9)	NS

**Table 2:** Mean number of items (±standard deviation) and proportion of exact, close, and far matches, as well as exclusions and intrusions.* 11 participants used e-CA and 11 others used a paper-based food record to evaluate 20 foods and drinks displayed on a table.

Naming of Foods and Beverages	e-CA * (*n* = 20 Items)	Paper-Based Food Record * (*n* = 20 Items)
Exact matches (%)	15.5 ± 2.2 (77.3%)	12.0 ± 3.3 (60.0%)
Close matches (%)	2.5 ± 1.2 (12.3%)	6.5 ± 2.8 (32.3%)
Far matches (%)	0.6 ± 1.0 (3.2%)	0.5 ± 0.7 (2.3%)
Exclusions (%)	1.4 ± 0.5 (6.8%)	1.1 ± 0.5 (5.5%)
Intrusions (%)	0 (0%)	0 (0%)

**Table 3:** Percentage of over or underestimation of the weight of displayed portions of 20 items using e-CA or a paper-based food record, analyzed by two independent investigators, and compared to real weight.

Foods and Beverages Displayed	Real Weight	e-CA	Paper-Based Food Record	
	(g)	(*n* = 11)	Investigator 1 (*n* = 11)	Investigator 2 (*n* = 11)
Breakfast cereal	80	−26%	−5%	19%
Bread	38	−3%	4%	−23%
Butter	12	+2%	59%	52%
Jam	31	−30%	−31%	−36%
Coffee	70	+130%	106%	114%
Cereal bar	19	−4%	43%	40%
Chicken + sauce	200	−38%	−26%	−31%
Rice	153	−35%	−4%	−17%
Carrots	165	−27%	−30%	−27%
Wine	150	−33%	−22%	−18%
Apple	150	−22%	−18%	−33%
Chocolate	17	+6%	10%	−1%
Yogurt	150	+2%	−5%	0
Cookies	28	+36%	17%	5%
Tea	200	+25%	−5%	5%
Quiche	166	+7%	−5%	−18%
Green salad	30	+14%	30%	50
Pumpkin soup	200	+55%	−5%	18%
Grapes	140	+1%	−38%	−10%
